# Transposon libraries identify novel *Mycobacterium bovis* BCG genes involved in the dynamic interactions required for BCG to persist during in vivo passage in cattle

**DOI:** 10.1186/s12864-019-5791-1

**Published:** 2019-05-28

**Authors:** Tom A. Mendum, Aneesh Chandran, Kerstin Williams, H. Martin Vordermeier, Bernardo Villarreal-Ramos, H. Wu, Albel Singh, Alex A. Smith, Rachel E. Butler, Aravind Prasad, Neeraj Bharti, Ruma Banerjee, Sunitha M. Kasibhatla, Apoorva Bhatt, Graham R. Stewart, Johnjoe McFadden

**Affiliations:** 1 School of Biosciences and Medicine, Faculty of Health and Medical Sciences, University of Surrey, Guildford, GU2 7XH UK; 2 Department of Bacteriology, Animal and Plant Health Agency, Weybridge, UK; 3 Institute of Microbiology and Infection, School of Biosciences, University of Birmingham, Edgbaston, Birmingham, UK; 4 HPC-Medical and Bioinformatics Applications Group, Centre for Development of Advanced Computing, Innovation Park, Panchavati, Pashan, Pune, Maharashtra 411008 India

**Keywords:** *Mycobacterium bovis* BCG, Transposon library, Cattle, *Mycobacterium tuberculosis*, Pyruvate carboxylase, Cyclopropane-fatty-acyl-phospholipid synthase, Rv1006

## Abstract

**Background:**

BCG is the most widely used vaccine of all time and remains the only licensed vaccine for use against tuberculosis in humans. BCG also protects other species such as cattle against tuberculosis, but due to its incompatibility with current tuberculin testing regimens remains unlicensed. BCG’s efficacy relates to its ability to persist in the host for weeks, months or even years after vaccination. It is unclear to what degree this ability to resist the host’s immune system is maintained by a dynamic interaction between the vaccine strain and its host as is the case for pathogenic mycobacteria.

**Results:**

To investigate this question, we constructed transposon mutant libraries in both BCG Pasteur and BCG Danish strains and inoculated them into bovine lymph nodes. Cattle are well suited to such an assay, as they are naturally susceptible to tuberculosis and are one of the few animal species for which a BCG vaccination program has been proposed. After three weeks, the BCG were recovered and the input and output libraries compared to identify mutants with in vivo fitness defects. Less than 10% of the mutated genes were identified as affecting in vivo fitness, they included genes encoding known mycobacterial virulence functions such as mycobactin synthesis, sugar transport, reductive sulphate assimilation, PDIM synthesis and cholesterol metabolism. Many other attenuating genes had not previously been recognised as having a virulence phenotype. To test these genes, we generated and characterised three knockout mutants that were predicted by transposon mutagenesis to be attenuating in vivo: pyruvate carboxylase, a hypothetical protein (BCG_1063), and a putative cyclopropane-fatty-acyl-phospholipid synthase. The knockout strains survived as well as wild type during in vitro culture and in bovine macrophages, yet demonstrated marked attenuation during passage in bovine lymph nodes confirming that they were indeed involved in persistence of BCG in the host.

**Conclusion:**

These data show that BCG is far from passive during its interaction with the host, rather it continues to employ its remaining virulence factors, to interact with the host’s innate immune system to allow it to persist, a property that is important for its protective efficacy.

**Electronic supplementary material:**

The online version of this article (10.1186/s12864-019-5791-1) contains supplementary material, which is available to authorized users.

## Background

*Mycobacterium bovis* BCG is the only vaccine, with proven efficacy against all three major mycobacterial pathogens, *M. tuberculosis* [1], *M. bovis* [2, 3] and *M. leprae* [4]. It is also the most widely administered vaccine of all time [5] with 90% of world’s children receiving BCG and over 120 million doses used each year. However, humans are not the only recipients. BCG vaccination has also been used to protect a variety of both wild and domestic species against *M. bovis*, including cattle and goats [6–9]. However, its widespread use in animals and in particular cattle, is compromised by the inability of the current tuberculin-based skin test regimens to distinguish *M. bovis* infection from BCG vaccination [9].

BCG was derived in the early 1900s by Calmette and Guérin, who sequentially subcultured an *M. bovis* isolate 230 times, until it lost the ability to cause disease in guinea pigs [8]. Although Calmette and Guérin’s BCG was attenuated, it still retained the ability to disseminate and survive in many infection models including guinea pigs, deer [7, 10] and cows (Vordermeier et al. Unpublished data). In humans, BCG can be recovered in skin biopsies weeks after inoculation [11] and has, on occasions, been re-isolated from individuals many years after vaccination [12, 13]. In immuno-compromised mice and humans, BCG retains sufficient residual virulence to cause disseminated disease [14, 15]. This is particularly apparent in HIV positive children [16, 17]. Studies in 2007 predicted that continued use of BCG in HIV-infected children would result in approximately 400 cases of disseminated BCG infection per 100,000 vaccination [18]; a finding that led to the World Health Organization (WHO) ceasing to recommend BCG vaccination for infants who show evidence of HIV infection [19]. A correlation between this residual virulence and BCG’s efficacy was proposed as long ago as 1952 [20] and has been confirmed in mice [21] where the ability to persist is associated with a prolonged protective immune response characterised by the presence of CD4 T_EM_ cells and an IFN-γ response [22]. Exactly how BCG is able to persist after vaccination is not clear. Is there a dynamic interplay with the host, in which its remaining virulence factors are used to resist innate killing mechanisms and to subvert the immune response in a manner similar to those observed with pathogenic mycobacteria, or are the bacilli more passive, remaining largely dormant in the host?

Genomic studies have revealed that the serial sub-culturing of BCG’s progenitor resulted in its loss of virulence and that later produced the distinct BCG strains used around the world, was associated with the sequential loss of several large segments of DNA and the acquisition of many SNPs, duplications and deletions, some of which have been shown to contribute to its attenuation [23, 24]. Yet BCG did not lose all of its virulence factors, and still retains functions such as cholesterol catabolism, resistance to oxidative and nitrosative stress, various lipid components (mycolic acids, phthiocerol dimycocerosate (PDIM), phenolic glycolipid (PGL), etc.) and others, that in *M. bovis* and the related *M. tuberculosis* are known to increase in vivo fitness [25]. Some of these may still contribute to the persistence of the vaccine in hosts such as cattle, and promote the induction of a protective immune response and vaccine efficacy. However, these same functions could also be involved in reactogenicity and the ability of BCG to cause disseminated disease in immunocompromised hosts. Indeed BCG strains derived from ‘later’ serial subcultures that have lost more of these virulence related functions than ‘early’ strains [26], are known to have a lower levels of reactogenicity [27] and to induce lower levels of the immune responses that correlate with protection [28]. Whether these differences between strains are sufficient to significantly alter the effectiveness of different BCG strains in either humans or animals remains difficult to determine [29, 30].

Transposon mutagenesis is a powerful tool to screen whole genomes for genes that confer a selectable trait, in this case virulence. However despite the utility of such an approach, relatively few genome-scale mycobacterial transposon libraries have been assessed in animal models [31, 32] and none in species that are naturally susceptible to tuberculosis. This is, in part, because of the difficulty of recovering sufficient mutants from a relevant animal model to make a genome scale assessment of fitness possible. A genome-wide transposon mutagenesis study was carried out by Sassetti et al [31] using an intravenous mouse model of *M. tuberculosis* infection and identified 194 virulence-associated genes, including the *mce*1 and *mce*4 operons and genes involved in biotin synthesis, lipid metabolism and trehalose recycling. Other studies using mice, guinea pigs or macaques [32–34] have used smaller pools of selected mutants and have identified virulence associated genes but not on a genome scale.

As part of a project to develop a diagnostic-compatible BCG vaccine, we initiated a transposon mutagenesis study to identify genes required for persistence of BCG in the bovine host. We utilized a recently developed model that allows the installation and recovery of large numbers of BCG following its injection into the prescapular lymph nodes of calves [35], enabling the recovery of a representative transposon library. Cattle are a particular relevant model, as they are naturally susceptible to tuberculosis and are one of the few species, other than humans, for which a BCG vaccination program has been proposed.

As BCG strains differ genetically and are known to induce different levels of immune responses we investigated two strains, BCG Pasteur 1173P2 and BCG Danish 1331. Both are ‘later’ strains, but from different BCG lineages [26]. BCG Pasteur 1173P2, is the strain recommended by WHO for animal efficacy studies [36] and has demonstrated efficacy in several animal trials, while BCG Danish 1331 is the only strain licensed in the UK for humans, and is considered to be the most likely strain to be used if a vaccination programme for cattle or wildlife were to be undertaken. Wedlock et al [37] compared the use of both strains in cattle and demonstrated no difference in protective efficacy, but did observe higher levels of IFNγ release from PPD (purified protein derivative) stimulated whole blood and larger skin test responses with BCG Pasteur.

By identifying the genetic components of BCG that are important for in vivo fitness we aim to identify the residual virulence factors that still operate in BCG and so describe the mechanisms by which BCG persists long enough to generate protective immunity.

## Results

### Construction of the BCG transposon library

The BCG Danish and BCG Pasteur libraries constructed by transduction with ɸMycoMarT7 contained 1.36 × 10^5^ cfu and 1.37 × 10^5^ cfu respectively. Sequencing of the transposon junctions and plots of accumulation curves predicted that these BCG Danish and Pasteur input libraries contained 4.7 × 10^4^ and 2.6 × 10^4^ unique transposon insertion (Additional file 1: Figure S1a and b) giving genomic coverage of 63 and 34% of all TA sites respectively. For BCG Danish this is likely to be a near saturated library as in *M. tuberculosis* 9% of possible insertion sites have non-permissive motifs and approximately 15% of possible insertion sites are in essential genes [38]. In both libraries, sites with non-permissive motifs had significantly lower rates of transposon insertion than other TA loci (6.1/5.6% of non-permissive sites had inserts while 60/34% of permissive site had inserts in BCG Danish/Pasteur, *p* < 0.0001).

### In vitro gene essentiality and fitness in BCG Pasteur and BCG Danish input libraries

In order to validate the libraries and to place the results from the animal passaged output libraries in context, we predicted the essentiality and relative fitness of genes in the in vitro input libraries using TRANSIT’s [49] Hidden Markov Model (HMM) and Resampling methods.

The HMM analysis is designed to identify genes that are predicted to be essential. When applied to the BCG Danish and Pasteur input libraries, the analysis predicted 651 and 643 genes to be essential respectively (Additional file 2: Data S1), with 440 in common between the two strains. Although this value appears low, many genes predicted to be essential in only one strain, were predicted to be growth-deficient in the other, consistent with them being severely growth-retarding. Of these essential genes, 77 and 61%, had previously been identified as essential in *M. tuberculosis* (based upon a fully saturated transposon library grown on 7H10 [38]), further validating the library construction.

We used the TRANSIT’s Resampling method (Additional file 3: Data S2) to identify genes for which transposon insertion abundance [40] differed significantly between the BCG Danish and BCG Pasteur input transposon libraries. Transposon insertions in seven genes were found to be significantly less abundant in BCG Danish than in BCG Pasteur when grown in vitro. These included three PDIM synthesis genes, *ppsA, ppsD and ppsE,* which were predicted to be growth advantaged in BCG Pasteur. In contrast, insertions in 240 genes were significantly less abundant in BCG Pasteur than in BCG Danish. These included *drrA, drrB* and *drrC*, which were predicted to be essential in BCG Pasteur but not Danish and the two-component regulator, *phoPR*; the *phoR* gene of BCG Danish is non-functional due to a frameshift at residue 91 (Additional file 1: Table S2) [26]. Many reductive sulfate assimilation genes, such as *sulP*, *cysA1, cysT, cysN, cysH* [24, 41] and *sirA*, were also predicted to be essential in BCG Pasteur but not Danish*.*

### The infection model

**Fig. 1:**
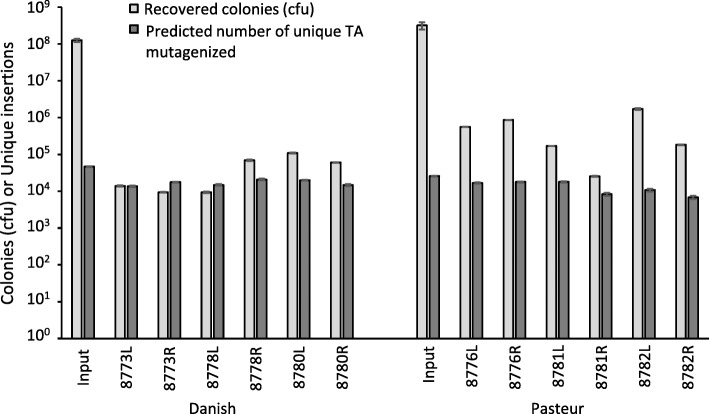
Numbers of BCG and the number of unique insert in the inoculating and recovered libraries. The predicted number of unique inserts were determined by plotting and extrapolating accumulation curves (Additional file 1: Figure S1a and b). Error bars represent standard errors for colony counts and root mean square errors for unique insert predictions

Aliquots of the frozen BCG Pasteur or BCG Danish input libraries containing 3.1 × 10^8^ cfu and 1.25 × 10^8^ cfu (Fig. 1) respectively, were injected into the left and the right prescapular lymph nodes of three calves. After 3 weeks, the nodes were excised and the BCG recovered. The nodes contained between 2.6 × 10^4^ and 1.7 × 10^6^ cfu, equating to libraries predicted to contain between 6.8 × 10^3^ to 4.7 × 10^4^ unique inserts (Fig. 1). These rates of recovery are similar to those observed previously in cattle [35] and in other infection models [10]. Differences in the percentage of bacilli recovered, and in the predicted numbers of unique inserts, in the two libraries were not significantly different, although recoveries for BCG Danish were generally lower than for BCG Pasteur. Neither was there a significant correlation in the number of BCG recovered from the left and right lymph nodes of individual animals, indicating the absence of systemic differences in BCG survival between individual calves. Nor was there a significant difference between the number of BCG recovered from the left, and from the right nodes, indicating and that there was no bias for survival for either of the nodes.

### In vivo gene fitness prediction in Pasteur and Danish libraries

When the BCG Danish and BCG Pasteur transposon libraries recovered from the lymph nodes of the cattle were compared to the inoculating input library, 274 and 100 genes respectively were determined to be attenuated in vivo (Additional file 2: Data S1). Notable functional gene groups included:

#### Mycobactin synthesis

**Fig. 2:**
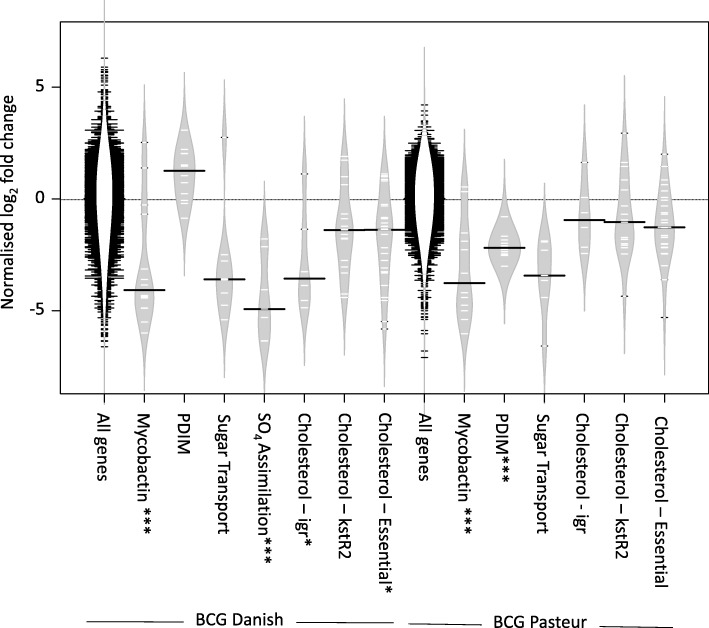
Bean plot of fold changes during in vivo passage in cattle for selected gene groups. White dashes represent fold changes for individual genes, black bars the median value of the group. The grey regions is a density plot of the data’s distribution. Values are normalised to the median value of the appropriate ‘All gene’ group. Essential cholesterol genes were those described by Griffin et al, 2011 [64] less the *mce*4 operon. The plots were created with BoxPlotR [80], *p* values are from hypergeometric tests, * < 0.05, ** < 0.01, *** < 0.001

Transposon insertion in many of the mycobactin synthesis genes *mbtABCDEFG* (BCG Danish) and *mbtBDEF* (BCG Pasteur) were strongly attenuating during passage in the cattle (*p* = 5 × 10^− 16^ and 0.0002 respectively, Fig. 2), indicating that iron uptake via mycobactin is important for persistence of BCG in the bovine lymph node.

#### Phthicerol dimycocerosates (PDIM) synthesis

Transposon inserts in genes involved in PDIM synthesis (*p* = 7 × 10^− 13^), notably *fadD26*, *fadD28*, *ppsABCDE*, *mas* and *papA5* were predicted to be attenuating in BCG Pasteur in vivo (Fig. 2); but were not significantly attenuating in BCG Danish. In the in vitro selected input library, PDIM synthesis genes were predicted to have a growth advantage *ie* they were over-represented in the BCG Pasteur input library but not in the Danish input library. These results indicated that the PDIM of the BCG Pasteur and BCG Danish libraries differed in their physiological functions both in vitro *and* in vivo*.* To investigate this phenomenon further, we compared PDIM extracted from BCG Pasteur and BCG Danish and observed that BCG Danish contained lower amounts of PDIM than BCG Pasteur (Additional file 1: Figure S2). This is likely due to a frameshift in PDIM synthesis gene, *ppsC* identified by Whole Genome Sequencing of the BCG isolates used to construct the library (Additional file 1: Table S2). This frameshift is liable to cause a change in the structure of the PDIM of BCG Danish. PDIMs are well established as important virulence factors in *M. bovis* and *M. tuberculosis* in mice [42] and guinea pigs [43]. These data demonstrate that PDIM also play a role in persistence of BCG in cattle; albeit one that is dependent on a complete synthesis of the lipid.

#### Reductive sulfate assimilation

**Fig. 3:**
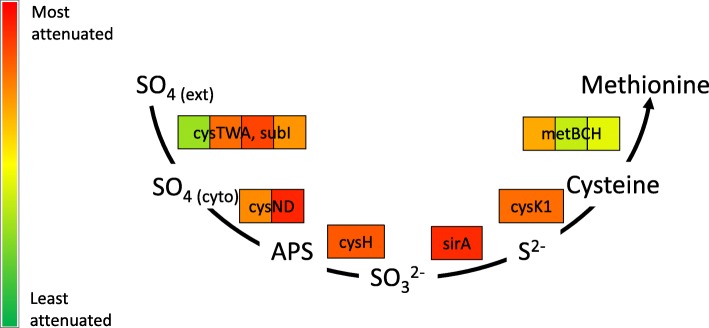
The assimilatory sulfate reduction pathway of BCG Danish. Gene names are coloured to indicate there log_2_ fold change during the BCG Danish in vivo passage in cattle. APS - Adenylyl-sulfate

Transposon inserts in genes involved in the reductive sulphur assimilation pathway from SO_4_ to cysteine, *eg cysA1*, *cysW*, *subI*, *cysN*, *sirA*, *cysK1* were predicted to be attenuating in BCG Danish (*p* = 2.5 × 10^− 5^, Figs. 2 and 3). In environments where reduced sulfur metabolites are unavailable this sulfate assimilation is important for the generation of sulphated metabolites such as amino acids, cofactors, etc. Of particular relevance are molecules such as mycothiol that regulate and counter oxidative stress [44]. In BCG Pasteur many of these genes were determined to be essential in vitro and so were absent from the input library.

#### Sugar transport

Transposon insertions in the genes associated with BCG’s two ABC sugar transport systems, the trehalose recycling system *sugABC*, *lpqY*, and BCG_2057c to BCG_2060c (*Rv2038c* to *Rv2041c*) were attenuating in vivo in both BCG Danish and in BCG Pasteur (Fig. 2). Insertions in the four genes that precede *sugABC*, BCG_1291c to BCG_1294 (equivalent to *Rv1231c*-*Rv1234)*, were also associated with loss of fitness, suggesting that these genes may also be involved in trehalose recycling.

#### Cholesterol metabolism

Transposon insertions in many genes involved in cholesterol catabolism, had significantly reduced fitness in the bovine lymph nodes for BCG Danish (Fig. 2, *p* = 0.016). A similar but non-significant effect was observed for BCG Pasteur. These included parts of the igr region [45] involved in the degradation of cholesterol’s side chains, and members of the kstR2 regulon [46] (*p* = 0.01 and 0.09). Interestingly, no fitness defect was detected for the genes of the cholesterol transporting *mce4* operon that was required for virulence of *M. tuberculosis* in mouse models [31, 47]. These data demonstrate that an ability to catabolise cholesterol remains important for BCG to survive in cattle, but that a cholesterol transport system other than mce4 may be operating.

### Identification of novel genes involved in BCG persistence

Although our data highlights the in vivo requirement for many previous recognised virulence factors, the majority of the genes identified as being involve in persistence of BCG in the bovine lymph node have not previously been ascribed fitness or virulence phenotypes. To confirm that these putative novel virulence factors are indeed involved in BCG persistence in vivo, we selected three genes that have not previously been recognised as being important for survival in animals, but that were predicted by these studies to be attenuating during in vivo passage. The genes selected were BCG_2988c (Rv2967c), BCG_1063 (Rv1006) and BCG_3780 (Rv3720). These encode respectively, pyruvate carboxylase, a hypothetical protein, and a possible cyclopropane-fatty-acyl-phospholipid synthase. Knockouts of these three genes were generated in BCG Danish using specialised transduction and confirmed by PCR and Sanger sequencing. As a control, a double knockout of BCG_3679c (Rv3615c, *espC*) and BCG_3680c (Rv3616, *espA*), genes that were not predicted by transposon analysis to contribute to in vivo fitness, was also generated.

**Fig. 4:**
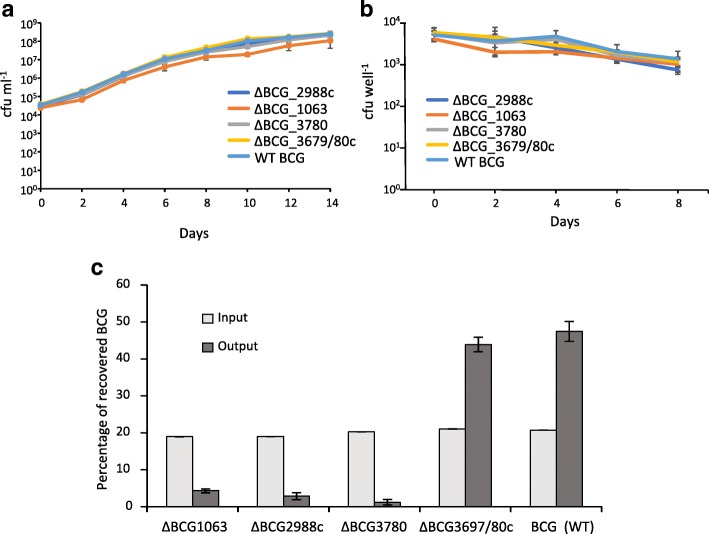
Competitive survival of selected mutants in vitro, ex vivo and in cattle. Competitive survival of selected mutants in (**a**) media, (**b**) bovine macrophages and during (**c**) in vivo survival in cattle. Approximately equal amounts of WT BCG Danish and selected knockouts were mixed and grown in media, infected into PBMC derived bovine macrophages, and inoculated into cattle lymph nodes. Inoculants and recovered BCG were enumerated on selective media. Cattle lymph nodes were resected after 3 weeks emulsified and BCG enumerated on selective media. Significance of the in vivo survival between of the three knockouts and the WT and control were all *p* < 0.001 using Tukey’s test. Error bars represent standard errors

We first determined whether the loss of the genes had a non-specific growth defect by tested their in vitro fitness, in a competition assay. When co-cultured with wild type BCG in 7H9 media none of the mutants showed a loss of fitness when compared to WT (Fig. 4a).

We next sought to determine whether the putative BCG persistence genes influenced their ability to survive the innate immune responses present in bovine macrophages by infecting peripheral blood mononuclear cell (PBMC) derived bovine macrophages with a mixture of WT BCG and the knockout (KO) mutants. None of the KO mutants were attenuated, when compared to the wild type in macrophages (Fig. 4b).

Finally, we sought to confirm the in vivo fitness phenotypes of the genes that were predicted by the transposon library data. KO mutants were mixed with wild type BCG Danish at approximately equal ratios and inoculated into the prescapular lymph nodes of 5 cattle using the same protocol as was used for the transposon library inoculation. After 3 weeks the mix of mutants and WT were recovered from the lymph nodes and enumerated on selective media. All of the knockouts showed significant attenuation except for the BCG_3615c/3616 control (Fig. 4c).

## Discussion

The ability of the BCG vaccine strain to persist in bovine lymph nodes involves both previously recognised virulence genes, as well as a large number of genes whose involvement in mycobacterial virulence has not so far been suspected. An examination of the gene groups identified as involved in this ability to persist in vivo reveals an interaction with the host that is far from passive, but rather is characterised by specific requirements that share commonality with the interactions that are known to occur during virulent mycobacterial infection. Notable amongst these fitness associated genes were groups associated with mycobactin synthesis, sulfur assimilation, PDIM synthesis, sugar transport and cholesterol degradation.

Transposon insertions in the mycobactin gene cluster and associated genes were highly attenuating in both BCG Pasteur and BCG Danish. Mycobactin is a siderophore used to acquire iron in low iron environments [48–50]. These genes are known virulence factors in *M. tuberculosis*: *ΔmtbE* mutants are severely attenuated in guinea pigs and induce lower levels of pathology [50], while Δ*mtbB* mutants are attenuated for replication in THP-1 macrophages [48].

Genes of the reductive sulfur assimilation pathway were also required for BCG’s persistence in vivo with inactivation of many of the pathway’s genes being attenuating in BCG Danish. The generation of sulfated metabolites, in particular cysteine, has a crucial role in the intracellular survival of *M. tuberculosis*, both for growth and to protect against redox disruption and oxidative stress via the production of mycothiol [51]. There are conflicting reports of the importance of this reductive sulfur assimilation pathway in mycobacteria. Knockouts of *cysH* have been shown to attenuate *M. tuberculosis* in immunocompetent mice [52], and genes of the pathway are upregulated in intracellular *M. tuberculosis* [53]. However, in BCG, *cysA* and *subI* mutants are not impaired for survival in mice [41, 54], suggesting that the bacillus is able to acquire reduced sulfur nutrients such a methionine from the murine host. In contrast, the requirement for a sulfate assimilation pathway in these cattle experiments implied that such reduced S compounds [41, 44, 54] were not present in sufficient quantities to compensate for the loss of gene function and so may leave mutants lacking the sulfate reducing pathway susceptible to oxidative stress [44]. The loss of fitness observed for knockouts in several other genes involved in resistance to oxidative stress, such as *katG*, *caeA* and *sodC,* all of which were severely attenuating in our model further suggests that the BCG bacilli experience oxidative stress in the cattle lymph nodes.

Mycolipids were clearly involved in the ability of BCG Pasteur to persist in the host with many of the genes required for PDIM and PGL production such as the *ppsABCDE* gene cluster, *tesA*, *mas*, *papA5*, *fadD26* and *fadD28*, all predicted to be attenuating in cattle. This attenuation was highlighted as many of these mutants were predicted to be growth advantaged in the input library, a phenomena observed previously in PDIM/PGL knockout mutants in *M. tuberculosis* and BCG [55]. A loss of PDIM is well documented as leading to a loss of virulence in *M. tuberculosis* and has been demonstrated to attenuate BCG Pasteur in immunocompromised SCID mice, and too lead to a loss of BCG’s protective efficacy in immunocompetent BALB/c mice [56]. An equivalent PDIM phenotype was not seen in BCG Danish mutants: they were neither attenuated in cattle lymph nodes, nor did they have a growth advantage in the in vitro grown input library. This is likely attributed to a spontaneously acquired frameshift mutation identified in the *ppsC* gene of the BCG Danish used in these experiments. Although this mutation did not abolish PDIM, it did reduce the relative amount. The frameshift is also likely to lead to an altered PDIM structure as it is predicted to abolish the ketoreductase domain of PpsC. A frameshift mutation in a similar location in the *ppsC* of *M. marinum* also reduced the amount of PDIM rather than abolishing production, but did lead to an increase in triacylglycerides (TAGs), the loss of a lipid species (as determined by thin layer chromatography), and to an increase in cell wall permeability [57]. Mutations leading to loss of PDIM in mycobacteria occur regular during in vitro culture [55] and have occurred independently during the development of several BCG strains, including BCG Japan, Moreau and Glaxo [27, 56], underlining the advantage of performing our analysis in two different BCG strains.

Sugar transport, or at least recycling, was also clearly involved in persistence of BCG in the bovine host. BCG has two ABC sugar transporters. The *sugABC*, *lpqY* system recycles trehalose that is released by the antigen85 complex during mycolic acid synthesis. This function is known to be important for virulence and was previously identified as involved in virulence in a *M. tuberculosis* transposon library screen using an intravenous mouse model of infection [31]. The predicted attenuation of mutants in the preceding genes suggests that these genes also have a role in trehalose recycling. Very little is known about BCG’s other ABC sugar transport system, BCG_2057c to BCG_2060c, with no previous reports of a virulence phenotype.

The ability to degrade cholesterol is a well-known virulence determinant for *M. tuberculosis* [58–60]. Sterol catabolism is thought to provide carbon for both growth and respiration [61], as well as providing a mechanism to manipulate the host cell’s immune response [62]. In BCG Danish, transposon insertions in many of the genes involved in cholesterol metabolism were found to be attenuating, in particular genes of the igr operon [63], which degrade the cholesterol side chain [45], and *hsaC* together with many of the genes of the *kstR2* regulon [46]. Many of these regions were also identified by Griffin et al [64] in a genome-scale transposon library based assessment of genes required for in vitro growth on cholesterol, but with the notable exception of the *mce4* region that encodes a cholesterol transporter. The absence of a phenotype for tranposon insertions in the *mce4* operon, yet clear attenuation when genes encoding enzymes of the cholesterol degradation pathways are inactivated, implies the existence of an alternative mechanism of acquiring cholesterol that is active in these cattle experiments. This conclusion is consistent with data that shows that *M. tuberculosis* Δ*mce4A* can continue to grow on cholesterol as a sole carbon source [65, 66], but contrasts with data from mice where a Δ*mce4A* mutant has been shown to be attenuated [31, 66].

Although many of the genes identified as being required for persistence of BCG in the bovine lymph node had previously been identified as virulence genes in a variety of other models, many others had not previously been associated with virulence. To investigate and validate the transposon-predicted in vitro, ex vivo and in vivo phenotype of three of these genes we carried out a competition experiments with KO BCG strains. All three knockout strains behaved similarly to wild type BCG during in vitro culture, and during survival in bovine macrophage, but were attenuated during passage in cattle lymph nodes. The role of these genes in virulence has not previously been suspected so we will now assess their possible roles.

Pyruvate carboxylase’s primary function is the fixation of CO_2_ to generate oxaloacetate from pyruvate and so replenish the TCA cycle [67]. Our data show that although this function is not required for growth in cultured cells, a result consistent with lack of a phenotype reported for *M. tuberculosis* Δ*pca* in human THP-1 cells [67], the genes is nevertheless required for persistence of BCG in cattle lymph nodes.

BCG_1063 is a hypothetical protein. Very little is known of its function. It is pseudogenised in *M. leprae*, but its phenotype has not been further investigated.

BCG_3780 is a possible cyclopropane-fatty-acyl-phospholipid synthase, a family of mycobacterial enzymes that add methyl groups to unsaturated fatty acids to generate the cyclopropanate rings found in lipids such as mycolic acids and phospholipids [68]. BCG contains several orthologs of BCG_3780, some of which can impact virulence by modifying the inflammatory response [69]. Individual gene KOs are associated with both attenuation [70] and hypervirulence [71]. The closest BCG_3780 ortholog in the BCG Pasteur genome is *ufaA1* [68], a gene that in *M. tuberculosis* methylates oleic acid to produce the immunomodulatory molecule, tuberculostearic acid [72]. It is possible that ΔBCG_3780c fitness phenotype is mediate via an as yet unknown fatty acid in a similar manner to its orthologs; or that it complements *ufaA1*, whose expression is downregulated in BCG Danish and BCG Pasteur due to a mutation in the start codon of its regulator *sigK* [73].

These data demonstrate that the mode of attenuation of these mutants was not simply due to generalised growth defects, or to an ability to survive the innate immune response of cultured macrophages, but rather is related to the multi-faceted environment found in whole animal lymph nodes.

## Conclusions

BCG is the most widely used vaccine in the world and yet the mechanisms by which it is able to survive in the host to provoke a long-lasting immune response are virtually unknown. In this study we use transposon mutagenesis to, for the first time, dissect this important capacity. We demonstrate that survival of BCG in the bovine host requires a large number of genes, some of which have been previously identified as being involved in virulence in pathogenic mycobacteria, while many more have no previously described virulence role. The genes identified indicate that the survival of BCG in the host involves specific interactions between the bacilli and the host’s immune system that share similarities with the pathogenic processes observed for virulent mycobacteria. We confirm that three of these novel virulence genes are indeed required for persistence in the host, but in ways that were not demonstrable by standard in vitro and ex vivo assays of virulence. Further investigation of the function of these genes may uncover novel aspects of pathogenesis that could be targeted by novel therapies or subunit vaccines; or may be used to engineer a new generation of safer and more effective live mycobacterial vaccines.

## Methods

### BCG culture

*Mycobacterium bovis* BCG Pasteur 1173P2 and *M. bovis* BCG Danish 1331 (Staten’s Serum Institut, batch 111013B) were grown at 37 °C on 7H11 with 10% OADC, or in 7H9 with 10% OADC and 0.05% Tween^®^80. For BCG Danish, growth on solid media was stimulated by ensuring a high titer inoculum was present in the Petri dish (this was not necessary for BCG Pasteur). When selection was required antibiotics were used at 25–50 μg ml^− 1^ for kanamycin, 50 μg ml^− 1^ for hygromycin, 25 μg ml^− 1^ for zeocin and 50 μg ml^− 1^ for apramycin. Both strains were Whole Genome Sequenced at over 30 fold coverage and aligned to the BCG Pasteur 1173P3 genome (NC_008769) and SNPS and Indels determined (MicrobesNG, http://www.microbesng.uk, UK).

### Transposon library construction

Transposon libraries were generated according to Long et al [74]. Briefly, 100 ml of late log *M. bovis* BCG culture was washed twice in MP buffer (50 mM Tris-HCl, pH 7.5, 150 mM NaCl, 10 mM MgSO_4_, 2 mM CaCl_2_) at 37 °C, and then incubated with more than 1 × 10^11^ pfu of ɸMycoMarT7 phage for either 4 h, or overnight. The suspension was recovered by centrifugation and plated on ten 15 cm plates, and the library enumerated by serial dilution. After 3–4 weeks the library was scraped from the plates and aliquots frozen at − 80 °C.

### Inoculation and recovery of tranposon libraries from cattle

A frozen aliquot of each transposon library was re-suspended overnight in 7H9 to allow it to disperse. Male, Holstein-Friesian calves of 4–6 months of age were obtained from UK farms located in bovine TB-free areas and were housed in appropriate BSL2 animal accommodation. Aliquots of each of the input libraries containing approximately 1 × 10^8^ cfu (OD_600_ ~ 1.0) were inoculated into both the left and right, prescapular lymph nodes of three calves, as described by Villarreal-Ramos et al [35]. After 3 weeks, the calves were euthanised and the lymph nodes were excised, surface sterilised and macerated in PBS. BCG were enumerated and the whole library (the output library) was plated on selective media in 15 cm Petri dishes. Correlation between BCG numbers recovered from left and right lymph nodes were assessed using Pearson’s correlation coefficient and the significance of differences in the number of BCG recovered from individual cattle, and between left and right lymph node were assessed by 2-way ANOVA without replication. Differences between the percentage recovery for BCG Danish and BCG Pasteur input were assessed with a t-test.

### Preparation and sequencing of recovered libraries

DNA from inoculating and selected transposon libraries was isolated using the enzymatic lysis protocol of Belise et al [75] and transposon junctions amplified using a modified version of the protocol described by Long et al [74]. Briefly, a 5 μg aliquot of the DNA was sheared in Covaris® Sono 7 machine with settings: Incident Power 105, Duty Factor 5%, 200 cycles/burst for 80 s. The ends were repaired, blunt ended, and ‘A’ overhangs added, using the NEB End-It™ and NEB A-tailing kits (Epicentre, UK). Equimolar amounts of linkers, Adap1 and Adap2 (Additional file 1: Table S1) were annealed by heating to 95 °C for 5 min in 50 μM MgCl_2_ and cooling slowly to room temperature. The annealed linkers were ligated to the ‘A’ tailed ends at × 100 molar excess with T4 DNA ligase for 2 h at room temperature. After each step the extract was cleaned using QIAquick^®^ PCR cleanup kits, with extra washes after the ligation step. Transposon junctions were amplified using primers, IS6 and MarA to MarE (Additional file 1: Table S1), cycle condition were 95 °C for 10 s, 58 °C for 10 s, 72 °C for 30 s. The primers were designed to have a P5-index that identifies the sample and a random P7-index to allow PCR-generated artefacts to be identified and removed from the data. Real-time PCRs using EvaGreen® were used to determine the minimum number of cycles required to amplify the transposon junctions, and so minimise PCR amplification artefacts. PCR products between 400 and 600 bp were size selected on agarose gels, and extracted with QIAquick^®^ Gel extraction kits. The PCR fragment sizes were determined and quantified using a Bioanalyser 2100 and a mix of equimolar quantities of each preparation sequenced using a HiSeq®2500 with a single sequence read and double index reads.

### Analysis of transposon-site data

Sequence data were demultiplexed using the P5-index reads, and quality controlled and aligned as described by Mendum et al [76]. An additional step removed artefactual amplicons generated by PCR amplification, which were identified as those reads having both identical P7-index reads and the same genomic insertion site. Reads were aligned to the *M. bovis* BCG Pasteur 1173P2 genome (NC_008769) using Bowtie [77] and the transposon counts determined. Gene essentiality in the input libraries was predicted using TRANSIT’s [39] HMM method but excluding TA sites with a non-permissive motif as identified previously in *M. tuberculosis* [38]. The significance of differences in insertion rates between sites with permissive and non-permissive motifs was assessed using a chi-squared test. Direct comparison between the Pasteur and Danish input libraries, and between the input libraries and the output libraries that were recovered from the cattle, were carried out using TRANSIT’s Resampling method [40] with TRR normalisations, and the non-permissive sites removed. Genes were deemed attenuated in cattle if they had a corrected *p* value (q-value) < 0.05 in at least 2/3 of the 6 lymph nodes (Danish/Pasteur respectively) and had an average fold change across all the lymph nodes of more than 10 fold (log_2_ = 3.32). The significance of gene group enrichments were determined with hypergeometric tests.

### Selection and construction of BCG Danish knockout mutants

Three genes that had not previously been ascribed virulence roles were chosen to test the tranposon-library based predictions. The genes were chosen to fulfil two roles: to confirm the tranposon predictions and to positively identify new virulence factors. We picked pyruvate carboxylase with a well-established function; a cyclopropane-fatty-acyl-phospholipid synthase, with a poorly annotated function and a hypothetical gene with no annotated function. These were ranked 131st, 23rd and 89th most attenuated in vivo in BCG Danish (by fold change) out of 274 attenuating genes. To produce the knockout strains we constructed zeomycin and kanamycin resistant derivatives of pYUB854 (Hyg) (Bardarov et al, 2002), pANE001 (Zeo) and pANE002 (Kan). An inverse PCR using primers pYUB_inv_F and pYUB_inv_R was used to amplify the plasmid backbone of pYUB854 less the hygromycin cassette, and to add *Nde*1 and *Mfe*1 restriction ends. Zeomycin and kanamycin cassettes were amplified from plasmids pNCMTB and pMV306 [78] respectively, using primers, Zeo_casset_F/R and Kan_casset_F/R containing *Nde*1 and *Mfe*1 restriction sites at their 3′ ends. The antibiotic cassettes were then cloned into the pYUB854 backbone to give pANE001 and pANE002, and the constructs confirmed by Sanger sequencing. Genomic sequence upstream (LF) and downstream (RF) of the genes to be knocked out were PCR amplified using primers BCG2988_RF_R, BCG2988RF_L, etc. and cloned either side of the antibiotic cassettes of the cosmids pYUB854, pANE001, pANE002 and the apramycin resistant cosmid p0004S (a gift from W. R. Jacobs Jr). Transducing phage were constructed and transduced into BCG using the pHAE159 mycobacteriophage-based method of specialized transduction (Bardarov et al, 2002). The knockouts were confirmed by PCR using primers outside of the upstream and downstream flanking regions both alone and in combination with antibiotic cassette specific primers, such that PCR products would only be obtained if the antibiotic cassettes were in the predicted configuration. Primer sequences are listed in Additional file 1: Table S1.

### In vitro competition assays

A mix containing approximately equal amounts of the four mutants and WT BCG Danish were inoculated into broth and cultured for 14 days. At selected time points the numbers of each mutant were determined by serially diluting onto selective media. Numbers of wild type BCG were estimated by subtracting the antibiotic resistant colony numbers from counts from plates without antibiotics. The assays were repeated three times.

### Bovine macrophage preparations and infections

Heparin-anticoagulated blood was collected from adult cows and the peripheral blood mononuclear cells (PBMCs) isolated using Ficoll-Histopaque density gradient centrifugation from which the monocytes were isolated using CD14 MicroBeads (Miltenyi Biotec). The monocytes were differentiated into macrophages in 24 well plates containing complete RPMI supplemented with 1% sodium pyruvate, 1% penicillin/streptomycin and 20 ng ml^− 1^ of macrophage colony-stimulating factor (Miltenyi Biotec). Fresh medium was added at day 3 before cells were infected on day 6, at an MOI of 1, with a mixed BCG culture containing approximately equal amounts of wild-type and of each of the individual knockouts. After 4 h, the infected cells were washed three times with PBS. The intracellular bacilli were harvested at different time points by lysing the cells with 0.1% Triton™ X-100. The mixed culture used for infection, and harvested intracellular bacilli were enumerated as described for the in vitro competition assay. The assays were repeated three times.

### Inoculation and recovery of selected knockouts from cattle

To test the in vivo phenotype of selected BCG knockouts both the left and right pre-scapular lymph nodes of 5 cattle were inoculated with a mix of ~ 1 × 10^8^ cfu containing approximately equal numbers of each mutant and the wild type in the same manner as described for the transposon libraries. After 3 weeks the cattle were euthanized and the left and right lymph nodes from each cow excised and pooled and macerated. BCG were enumerated as described for the macrophages. Significance between the percentage abundance of each mutant in the recovered BCG were determined by One-way ANOVA and Tukey’s post-hoc test.

### Lipid analysis

Lipids were extracted from mycobacterial cells in three fractions as described by Dobson et al [79], with a few modifications. Briefly, free lipids from dried pellets were extracted with two consecutive extractions of 4 ml of petroleum ether (60–80 °C), and dried. Then, 2 ml of CH_3_OH: 0.3% NaCl (10:1, v/v) were added to the remaining pellets. To extract apolar lipids, two sequential extractions with 2 ml of petroleum ether (60–80 °C) were carried out, and upper organic layers were dried. For polar lipid extraction, 2.3 ml of CHCl_3_/CH_3_OH/0.3% NaCl (9:10:3, v/v/v) was added to the previous lower layer. The resulting supernatant was collected in another tube along with the supernatants obtained after washing the remaining pellets twice with CHCl_3_/CH_3_OH/0.3% NaCl (5:10:4, v/v/v). Equal volumes of CHCl_3_ and 0.3% NaCl were then added, and the lower phase was removed and dried. All lipids were resuspended in 200 μl of CHCl_3_/CH_3_OH (2:1, v/v). Apolar lipids were separated by 2D TLC using petroleum ether/ethyl acetate 98:2, followed by petroleum ether/acetone 98:2 and visualised by charring after staining with 5% molybdophosphoric acid.

## Additional files

Additional file 1: **Supplementary Tables and Figures.** Genomic differences between the strains, accumulation plots from which library sizes were estimated, primer sequences and lipid analysis. (DOCX 1109 kb)

Additional file 2: The predicted essentiality and in vivo fitness of BCG genes. Output from the comparisons between the input and output libraries, describing the relative fitness of each gene during passage through the cattle. (XLSX 2251 kb)

Additional file 3: Differences in in vitro fitness between BCG Danish and BCG Pasteur input libraries. Output from the genomic comparisons of the two input libraries describing relative in vitro fitness between BCG Danish and BCG Pasteur. (XLSX 241 kb)

## Abbreviations

BCG: *Mycobacterium bovis* Bacillus Calmette–Guérin
HIV: Human Immunodeficiency Virus
HMM: Hidden Markov Model
KO: Knockout
PBMC: Peripheral Blood Monocyte
PDIM: phthiocerol dimycocerosate
PGL: Phenolic Glycolipids
PPD: Purified Protein Derivative
TAG: Triacyl Glyceride
TCA: Tricarboxylic Acid
WHO: World Health Organization

## References

[1] Rodrigues LC, Diwan VK, Wheeler JG (1993). Protective effect of BCG against tuberculous meningitis and miliary tuberculosis: a meta-analysis. Int J Epidemiol.

[2] Ameni G, Tafess K, Zewde A, Eguale T, Tilahun M, Hailu T, Sirak A, Salguero FJ, Berg S, Aseffa A (2018). Vaccination of calves with Mycobacterium bovis Bacillus Calmette-Guerin reduces the frequency and severity of lesions of bovine tuberculosis under a natural transmission setting in Ethiopia. Transbound Emerg Dis.

[3] Hope JC, Thom ML, Villarreal-Ramos B, Vordermeier HM, Hewinson RG, Howard CJ (2005). Vaccination of neonatal calves with Mycobacterium bovis BCG induces protection against intranasal challenge with virulent M. bovis. Clin Exp Immunol.

[4] Stanley SJ, Howland C, Stone MM, Sutherland I (1981). BCG vaccination of children against leprosy in Uganda: final results. J Hyg.

[5] McShane H (2011). Tuberculosis vaccines: beyond bacille Calmette–Guérin. Philos Trans Royal Soc B: Biol Sci.

[6] Chambers MA, Rogers F, Delahay RJ, Lesellier S, Ashford R, Dalley D, Gowtage S, Dave D, Palmer S, Brewer J (2011). Bacillus Calmette-Guerin vaccination reduces the severity and progression of tuberculosis in badgers. Proc Biol Sci.

[7] Palmer MV, Thacker TC, Waters WR (2009). Vaccination with Mycobacterium bovis BCG strains Danish and Pasteur in white-tailed deer (Odocoileus virginianus) experimentally challenged with Mycobacterium bovis. Zoonoses Public Health.

[8] Calmette A (1931). Preventive vaccination against tuberculosis with BCG. Proc Royal Soc Med.

[9] Vordermeier HM, Jones GJ, Buddle BM, Hewinson RG, Villarreal-Ramos B (2016). Bovine tuberculosis in cattle: vaccines, DIVA tests, and host biomarker discovery. Annual Rev Animal Biosci.

[10] Slobbe L, Lockhart E, O'Donnell MA, Mackintosh C, De Lisle G, Buchan G (1999). An in vivo comparison of bacillus Calmette–Guérin (BCG) and cytokine-secreting BCG vaccines. Immunology.

[11] Minassian AM, Satti I, Poulton ID, Meyer J, Hill AV, McShane H (2012). A human challenge model for Mycobacterium tuberculosis using Mycobacterium bovis bacille Calmette-Guerin. J Infect Dis.

[12] Seishima M, Fujisawa T, Yamanaka S (2006). Bcg granuloma appearing more than 50 years after vaccination. Arch Dermatol.

[13] Ameeruddin NU, Luke Elizabeth H (2014). Impact of isoniazid resistance on virulence of global and south Indian clinical isolates of Mycobacterium tuberculosis. Tuberculosis (Edinb).

[14] Talbot EA, Perkins MD, Silva SF, Frothingham R (1997). Disseminated bacille Calmette-Guerin disease after vaccination: case report and review. Clin Infect Dis.

[15] Milstien JB, Gibson JJ (1990). Quality control of BCG vaccine by WHO: a review of factors that may influence vaccine effectiveness and safety. Bull World Health Organ.

[16] Hesseling AC, Schaaf HS, Hanekom WA, Beyers N, Cotton MF, Gie RP, Marais BJ, van Helden P, Warren RM (2003). Danish bacille Calmette-Guerin vaccine-induced disease in human immunodeficiency virus-infected children. Clin Infect Dis.

[17] Hesseling AC, Rabie H, Marais BJ, Manders M, Lips M, Schaaf HS, Gie RP, Cotton MF, van Helden PD, Warren RM (2006). Bacille Calmette-Guerin vaccine-induced disease in HIV-infected and HIV-uninfected children. Clin Infect Dis.

[18] Hesseling AC, Marais BJ, Gie RP, Schaaf HS, Fine PE, Godfrey-Faussett P, Beyers N (2007). The risk of disseminated Bacille Calmette-Guerin (BCG) disease in HIV-infected children. Vaccine.

[19] WHO (2007). Revised BCG vaccination guidelines for infants at risk for HIV infection. Wkly Epidemiol Rec.

[20] Dubos RaD J. The white plague: tuberculosis. Man Soc. 1952;163.

[21] Zhang L, Ru HW, Chen FZ, Jin CY, Sun RF, Fan XY, Guo M, Mai JT, Xu WX, Lin QX (2016). Variable virulence and efficacy of BCG vaccine strains in mice and correlation with genome polymorphisms. Mol Ther.

[22] Kaveh DA, Garcia-Pelayo MC, Hogarth PJ (2014). Persistent BCG bacilli perpetuate CD4 T effector memory and optimal protection against tuberculosis. Vaccine.

[23] Hsu T, Hingley-Wilson SM, Chen B, Chen M, Dai AZ, Morin PM, Marks CB, Padiyar J, Goulding C, Gingery M (2003). The primary mechanism of attenuation of bacillus Calmette-Guerin is a loss of secreted lytic function required for invasion of lung interstitial tissue. Proc Natl Acad Sci U S A.

[24] Abdallah AM, Hill-Cawthorne GA, Otto TD, Coll F, Guerra-Assuncao JA, Gao G, Naeem R, Ansari H, Malas TB, Adroub SA (2015). Genomic expression catalogue of a global collection of BCG vaccine strains show evidence for highly diverged metabolic and cell-wall adaptations. Sci Rep.

[25] Forrellad MA, Klepp LI, Gioffré A, Sabio y García J, Morbidoni HR, Santangelo MP, Cataldi AA, Bigi F (2013). Virulence factors of the Mycobacterium tuberculosis complex. Virulence.

[26] Brosch R, Gordon SV, Garnier T, Eiglmeier K, Frigui W, Valenti P, Dos Santos S, Duthoy S, Lacroix C, Garcia-Pelayo C (2007). Genome plasticity of BCG and impact on vaccine efficacy. Proc Natl Acad Sci U S A.

[27] Chen JM, Islam ST, Ren H, Liu J (2007). Differential productions of lipid virulence factors among BCG vaccine strains and implications on BCG safety. Vaccine.

[28] Davids V, Hanekom WA, Mansoor N, Gamieldien H, Sebastian JG, Hawkridge A, Hussey GD, Hughes EJ, Soler J, Murray RA (2006). The effect of Bacille Calmette-Guérin vaccine strain and route of administration on induced immune responses in vaccinated infants. J Infect Dis.

[29] Dockrell HM, Smith SG (2017). What have we learnt about BCG vaccination in the last 20 years?. Front Immunol.

[30] Mangtani P, Abubakar I, Ariti C, Beynon R, Pimpin L, Fine PEM, Rodrigues LC, Smith PG, Lipman M, Whiting PF (2014). Protection by BCG vaccine against tuberculosis: a systematic review of randomized controlled trials. Clin Infect Dis.

[31] Sassetti CM, Rubin EJ (2003). Genetic requirements for mycobacterial survival during infection. Proc Natl Acad Sci U S A.

[32] Dutta NK, Mehra S, Didier PJ, Roy CJ, Doyle LA, Alvarez X, Ratterree M, Be NA, Lamichhane G, Jain SK (2010). Genetic requirements for the survival of tubercle bacilli in primates. J Infect Dis.

[33] Jain SK, Hernandez-Abanto SM, Cheng QJ, Singh P, Ly LH, Klinkenberg LG, Morrison NE, Converse PJ, Nuermberger E, Grosset J (2007). Accelerated detection of Mycobacterium tuberculosis genes essential for bacterial survival in Guinea pigs, compared with mice. J Infect Dis.

[34] Lamichhane G, Tyagi S, Bishai WR (2005). Designer arrays for defined mutant analysis to detect genes essential for survival of Mycobacterium tuberculosis in mouse lungs. Infect Immun.

[35] Villarreal-Ramos B, Berg S, Chamberlain L, McShane H, Hewinson RG, Clifford D, Vordermeier M (2014). Development of a BCG challenge model for the testing of vaccine candidates against tuberculosis in cattle. Vaccine.

[36] WHO: report of WHO/FAO/OIE consultation on animal tuberculosis. 1994, WHO/CDS/VPH/94.

[37] Wedlock DN, Denis M, Vordermeier HM, Hewinson RG, Buddle BM (2007). Vaccination of cattle with Danish and Pasteur strains of Mycobacterium bovis BCG induce different levels of IFNγ post-vaccination, but induce similar levels of protection against bovine tuberculosis. Vet Immunol Immunopathol.

[38] DeJesus MA, Gerrick ER, Xu W, Park SW, Long JE, Boutte CC, Rubin EJ, Schnappinger D, Ehrt S, Fortune SM, et al. Comprehensive essentiality analysis of the Mycobacterium tuberculosis genome via saturating transposon mutagenesis. mBio. 2017;8(1). 10.1128/mBio.02133-16 PMC5241402 28096490

[39] DeJesus MA, Ambadipudi C, Baker R, Sassetti C, Ioerger TR (2015). TRANSIT--A software tool for Himar1 TnSeq analysis. PLoS Comput Biol.

[40] Carey AF, Rock JM, Krieger IV, Chase MR, Fernandez-Suarez M, Gagneux S, Sacchettini JC, Ioerger TR, Fortune SM (2018). TnSeq of Mycobacterium tuberculosis clinical isolates reveals strain-specific antibiotic liabilities. PLoS Pathog.

[41] Wooff E, Michell SL, Gordon SV, Chambers MA, Bardarov S, Jacobs WR, Hewinson RG, Wheeler PR (2002). Functional genomics reveals the sole sulphate transporter of the Mycobacterium tuberculosis complex and its relevance to the acquisition of Sulphur in vivo. Mol Microbiol.

[42] Cox JS, Chen B, McNeil M, Jacobs WR (1999). Complex lipid determines tissue-specific replication of Mycobacterium tuberculosis in mice. Nature.

[43] Hotter GS, Wards BJ, Mouat P, Besra GS, Gomes J, Singh M, Bassett S, Kawakami P, Wheeler PR, de Lisle GW (2005). Transposon mutagenesis of Mb0100 at the ppe1-nrp locus in Mycobacterium bovis disrupts phthiocerol dimycocerosate (PDIM) and glycosylphenol-PDIM biosynthesis, producing an avirulent strain with vaccine properties at least equal to those of M. bovis BCG. J Bacteriol.

[44] Berney M, Berney-Meyer L, Wong KW, Chen B, Chen M, Kim J, Wang J, Harris D, Parkhill J, Chan J (2015). Essential roles of methionine and S-adenosylmethionine in the autarkic lifestyle of Mycobacterium tuberculosis. Proc Natl Acad Sci U S A.

[45] Chang JC, Miner MD, Pandey AK, Gill WP, Harik NS, Sassetti CM, Sherman DR (2009). Igr genes and Mycobacterium tuberculosis cholesterol metabolism. J Bacteriol.

[46] Kendall SL, Burgess P, Balhana R, Withers M, Ten Bokum A, Lott JS, Gao C, Uhia-Castro I, Stoker NG (2010). Cholesterol utilization in mycobacteria is controlled by two TetR-type transcriptional regulators: kstR and kstR2. Microbiology (Reading, England).

[47] Senaratne RH, Sidders B, Sequeira P, Saunders G, Dunphy K, Marjanovic O, Reader JR, Lima P, Chan S, Kendall S (2008). Mycobacterium tuberculosis strains disrupted in mce3 and mce4 operons are attenuated in mice. J Med Microbiol.

[48] De Voss JJ, Rutter K, Schroeder BG, Su H, Zhu Y, Barry CE (2000). The salicylate-derived mycobactin siderophores of Mycobacterium tuberculosis are essential for growth in macrophages. Proc Natl Acad Sci U S A.

[49] Madigan CA, Martinot AJ, Wei JR, Madduri A, Cheng TY, Young DC, Layre E, Murry JP, Rubin EJ, Moody DB (2015). Lipidomic analysis links mycobactin synthase K to iron uptake and virulence in M. tuberculosis. PLoS Pathog.

[50] Reddy PV, Puri RV, Chauhan P, Kar R, Rohilla A, Khera A, Tyagi AK (2013). Disruption of mycobactin biosynthesis leads to attenuation of Mycobacterium tuberculosis for growth and virulence. J Infect Dis.

[51] Newton GL, Fahey RC (2002). Mycothiol biochemistry. Arch Microbiol.

[52] Senaratne RH, Mougous JD, Reader JR, Williams SJ, Zhang T, Bertozzi CR, Riley LW (2007). Vaccine efficacy of an attenuated but persistent Mycobacterium tuberculosis cysH mutant. J Med Microbiol.

[53] Parvati Sai Arun PV, Miryala SK, Rana A, Kurukuti S, Akhter Y, Yellaboina S (2018). System-wide coordinates of higher order functions in host-pathogen environment upon Mycobacterium tuberculosis infection. Sci Rep.

[54] McAdam RA, Weisbrod TR, Martin J, Scuderi JD, Brown AM, Cirillo JD, Bloom BR, Jacobs WR, Jr.: In vivo growth characteristics of leucine and methionine auxotrophic mutants of Mycobacterium bovis BCG generated by transposon mutagenesis. Infect Immun 1995, 63(3):1004–1012. 10.1128/iai.63.3.1004-1012.1995 PMC173102 7868221

[55] Domenech P, Reed MB (2009). Rapid and spontaneous loss of phthiocerol dimycocerosate (PDIM) from Mycobacterium tuberculosis grown in vitro: implications for virulence studies. Microbiology.

[56] Tran V, Ahn SK, Ng M, Li M, Liu J (2016). Loss of lipid virulence factors reduces the efficacy of the BCG vaccine. Sci Rep.

[57] Williams EA, Mba Medie F, Bosserman RE, Johnson BK, Reyna C, Ferrell MJ, Champion MM, Abramovitch RB, Champion PA. A nonsense mutation in Mycobacterium marinum that is suppressible by a novel mechanism. Infect Immun. 2017;85(2). 10.1128/IAI.00653-16 PMC5278160 27789543

[58] Yam KC, D'Angelo I, Kalscheuer R, Zhu H, Wang JX, Snieckus V, Ly LH, Converse PJ, Jacobs WR, Strynadka N (2009). Studies of a ring-cleaving dioxygenase illuminate the role of cholesterol metabolism in the pathogenesis of Mycobacterium tuberculosis. PLoS Pathog.

[59] Hu Y, van der Geize R, Besra GS, Gurcha SS, Liu A, Rohde M, Singh M, Coates A (2010). 3-Ketosteroid 9alpha-hydroxylase is an essential factor in the pathogenesis of Mycobacterium tuberculosis. Mol Microbiol.

[60] Nesbitt NM, Yang X, Fontan P, Kolesnikova I, Smith I, Sampson NS, Dubnau E (2010). A thiolase of Mycobacterium tuberculosis is required for virulence and production of androstenedione and androstadienedione from cholesterol. Infect Immun.

[61] Brzezinska M, Szulc I, Brzostek A, Klink M, Kielbik M, Sulowska Z, Pawelczyk J, Dziadek J (2013). The role of 3-ketosteroid 1(2)-dehydrogenase in the pathogenicity of Mycobacterium tuberculosis. BMC Microbiol.

[62] Bandyopadhyay U, Chadha A, Gupta P, Tiwari B, Bhattacharyya K, Popli S, Raman R, Brahamachari V, Singh Y, Malhotra P (2017). Suppression of toll-like receptor 2-mediated proinflammatory responses by Mycobacterium tuberculosis protein Rv3529c. J Leukoc Biol.

[63] Chang JC, Harik NS, Liao RP, Sherman DR (2007). Identification of mycobacterial genes that alter growth and pathology in macrophages and in mice. J Infect Dis.

[64] Griffin JE, Gawronski JD, Dejesus MA, Ioerger TR, Akerley BJ, Sassetti CM (2011). High-resolution phenotypic profiling defines genes essential for mycobacterial growth and cholesterol catabolism. PLoS Pathog.

[65] Singh P, Sinha R, Tyagi G, Sharma NK, Saini NK, Chandolia A, Prasad AK, Varma-Basil M, Bose M (2018). PDIM and SL1 accumulation in Mycobacterium tuberculosis is associated with mce4A expression. Gene.

[66] Pandey AK, Sassetti CM (2008). Mycobacterial persistence requires the utilization of host cholesterol. Proc Natl Acad Sci U S A.

[67] Basu P, Sandhu N, Bhatt A, Singh A, Balhana R, Gobe I, Crowhurst NA, Mendum TA, Gao L, Ward JL (2018). The anaplerotic node is essential for the intracellular survival of Mycobacterium tuberculosis. J Biol Chem.

[68] Meena LS, Chopra P, Vishwakarma RA, Singh Y (2013). Biochemical characterization of an S-adenosyl-l-methionine-dependent methyltransferase (Rv0469) of Mycobacterium tuberculosis. Biol Chem.

[69] Barkan D, Hedhli D, Yan HG, Huygen K, Glickman MS (2012). Mycobacterium tuberculosis lacking all mycolic acid cyclopropanation is viable but highly attenuated and hyperinflammatory in mice. Infect Immun.

[70] Glickman MS, Cox JS, Jacobs WR (2000). A novel mycolic acid cyclopropane synthetase is required for cording, persistence, and virulence of Mycobacterium tuberculosis. Mol Cell.

[71] Rao V, Gao F, Chen B, Jacobs WR, Glickman MS (2006). Trans-cyclopropanation of mycolic acids on trehalose dimycolate suppresses Mycobacterium tuberculosis -induced inflammation and virulence. J Clin Invest.

[72] Nuzzo I, Galdiero M, Bentivoglio C, Galdiero R, Romano Carratelli C (2002). Apoptosis modulation by mycolic acid, tuberculostearic acid and trehalose 6,6′-dimycolate. J Infect.

[73] Charlet D, Mostowy S, Alexander D, Sit L, Wiker HG, Behr MA (2005). Reduced expression of antigenic proteins MPB70 and MPB83 in Mycobacterium bovis BCG strains due to a start codon mutation in sigK. Mol Microbiol.

[74] Long JE, DeJesus M, Ward D, Baker RE, Ioerger T, Sassetti CM (2015). Identifying essential genes in Mycobacterium tuberculosis by global phenotypic profiling. Methods in molecular biology (Clifton, NJ).

[75] Belisle JTMS, Hill PJ, Parish T BA (2008). Isolation of Mycobacterium species genomic DNA. Mycobacteria Protocols.

[76] Mendum TA, Wu H, Kierzek AM, Stewart GR (2015). Lipid metabolism and type VII secretion systems dominate the genome scale virulence profile of Mycobacterium tuberculosis in human dendritic cells. BMC Genomics.

[77] Langmead B, Trapnell C, Pop M, Salzberg SL (2009). Ultrafast and memory-efficient alignment of short DNA sequences to the human genome. Genome Biol.

[78] Andreu N, Zelmer A, Fletcher T, Elkington PT, Ward TH, Ripoll J, Parish T, Bancroft GJ, Schaible U, Robertson BD (2010). Optimisation of bioluminescent reporters for use with mycobacteria. PLoS One.

[79] Dobson G MD, Minnikin SM, Parlett JH, Goodfellow M, Ridell M, Magnusson M: Systematic analysis of complex mycobacterial lipids. In: *Chemical methods in bacterial systematics* Edited by Goodfellow M MD. London (UK): Academic Press; 1985: 237–265.

[80] Spitzer M, Wildenhain J, Rappsilber J, Tyers M (2014). BoxPlotR: a web tool for generation of box plots. Nat Methods.

