# Supplementary material for: Transposon libraries identify novel Mycobacterium bovis BCG genes involved in the dynamic interactions required for BCG to persist during in vivo passage in cattle
Source: BMC Genomics. 2019 May 28;20:431. doi: 10.1186/s12864-019-5791-1 (PMC6540422; doi:10.1186/s12864-019-5791-1)
Supplement: Supplementary file 1 — Supplementary Tables and Figures. Genomic differences between the strains, accumulation plots from which library sizes were estimated, primer sequences and lipid analysis. (DOCX 1109 kb) [file 12864_2019_5791_MOESM1_ESM.docx]

**Transposon mutagenesis identifies novel *Mycobacterium bovis* BCG genes involved in a dynamic interactions between BCG vaccine and bovine host.**

**Mendum TA, Chandran A, Williams K *et al.***

**Additional File 1. Supplementary Tables and Figures.** Tables of primers, genome comparisons, plots from library sizes were estimated and lipid analysis

| Adap1 | **caagcAGAAGACGGCATACGAGAT**NNNNNNNNGTGACTGGAGTTCAGACGTGTGCTCTTCCgatct |
| --- | --- |
| Adap2 | gatcgGAAgagca-PHO |
| MarA | **AATGATACGGCGACCACCGAGATCTACACTGTTCCGA**ACACTCTTTCCCTACACGACGCTCTTCCGATCT**CGGGGACTTATCAGCCAACC** |
| MarB | **AATGATACGGCGACCACCGAGATCTACACTTCCGGAG**ACACTCTTTCCCTACACGACGCTCTTCCGATCT**TCGGGGACTTATCAGCCAACC** |
| MarC | **AATGATACGGCGACCACCGAGATCTACACGCCGATGT**ACACTCTTTCCCTACACGACGCTCTTCCGATCT**GATACGGGGACTTATCAGCCAACC** |
| MarD | **AATGATACGGCGACCACCGAGATCTACACCATGATCG**ACACTCTTTCCCTACACGACGCTCTTCCGATCT**TATCTACGGGGACTTATCAGCCAACC** |
| MarE | **AATGATACGGCGACCACCGAGATCTACACCGCGCGGT**ACACTCTTTCCCTACACGACGCTCTTCCGATCT**CGGGGACTTATCAGCCAACC** |
| MarF | **AATGATACGGCGACCACCGAGATCTACACACACGATC**ACACTCTTTCCCTACACGACGCTCTTCCGATCT**TCGGGGACTTATCAGCCAACC** |
| MarG | **AATGATACGGCGACCACCGAGATCTACACAAGTAGAG**ACACTCTTTCCCTACACGACGCTCTTCCGATCT**GATACGGGGACTTATCAGCCAACC** |
| Zeo_casset_F | GAACTCCAATTGATGGCCAAGTTGACCAGTGC |
| Zeo_casset_R | GAACTCCATATGTCAGTCCTGCTCCTCGGCCAC |
| Kan_casset_F | GAACTCCAATTGATGAGCCATATTCAACGGG |
| Kan_casset_R | CGAAACCATATGTTAGAAAAACTCATCGAGC |
| pYUB_inv_F | GACATC CAATTGTCACAGCGGACCTCTATTC |
| pYUB_inv_R | GATCTCCATATGAACTGGCGCAGTTCCTCTGG |
| BCG2988_RF_R | TTGCAGGGTTGACCAGGATG |

| BCG2988_RF_F | GATCTCAAGCTTTGTTGGTGGTGGTGAGCTG |
| --- | --- |
| BCG2988_RF_R | GATCTCACTAGTTTGCAGGGTTGACCAGGATG |
| BCG2988_LF_F | GATCTCCTTAAGTGCATCAACAGCGGCAAGTAC |
| BCG2988_LF_R | GATCTCTCTAGACCTTGGAAAACACGTGTGGC |
| BCG3780_RF_F | GATCTCAAGCTTGCCCTAGGCGTTGTCTATCC |
| BCG3780_RF_R | GATCTCACTAGTTTTCGCTACCGATCTGCTGG |
| BCG3780_LF_F | GATCTCCTTAAGTCGTATGGGACACTGGGCTA |
| BCG3780_LF_R | GATCTCTCTAGACTGAGTCTGCCCGTTGTCATC |
| BCG1063_RF_F | TTTTTTTTCCATAGATTGGACGCTTCGTAGGTGGCTTTC |
| BCG1063_RF_R | TTTTTTTTCCATCTTTTGGTCGGCAAGGACATCATCAGG |
| BCG1063_LF_F | TTTTTTTTCCATAAATTGGGATAGCTTCCAGACACGCCA |
| BCG1063_LF_R | TTTTTTTTCCATTTCTTGGAAGATCACACCGAGCGTCAA |
| BCG3645/46_RF_F | GATCTCAAGCTTCCTGACCACGTTTGCTGC |
| BCG3645/46_RF_R | GATCTCACTAGTCGTGCTCTATTAATGCTG |
| BCG3645/46_LF_F | GATCTCCTTAAGTCTATCAGTAGGCGGCTAG |
| BCG3645/46_LF_R | GATCTCTCTAGAAACTGCGCTGCGACAATG |

| BCG1063_RF_CHK_F | AAGCCGACAATCCACCGCTG |  |
| --- | --- | --- |
| BCG1063_RF_CHK_R | GACCAATTACCTGACCGGGG |  |
| BCG1063_LF_CHK_F | ACGCACGCTTGATAGACCTC |  |
| *BCG1063_LF_CHK_R* | *GAGTCGCTTAACGGTGTCCA* | |
| *BCG2988_RF_CHK_F* | *GGTCGTGTCCACGAACTTCC* | |
| BCG2988_RF_CHK_R | GTCGAGGTCGAACATGCCCG |  |
| BCG2988_LF_CHK_F | GCAAGGATTTCCCCGACAAC |  |
| BCG2988_LF_CHK_R | AAGTCGTCCTCCACGAAGTC |  |
| BCG3780_RF_CHK__F | CCATGCGCCAGAGTTGTTTC |  |
| BCG3780_RF_CHK_R | GGGCGATCGAATCGGTGAT |  |
| BCG3780_LF_CHK_F | AGAACTTCTCACCGTCTCGC |  |
| BCG3780_LF_CHK_R | TATCGGTCTGCGATTCCGAC |  |
| BCG3645/46_RF_ CHK_ F | AAATCGCGTACGTGG |  |
| BCG3645/46_ RF_CHK_R | GAAGTGCACGCAGTTGCC |  |
| BCG3645/46_LF_CHK_F | CAAGTTGACCAGTGCCGTTC |  |
| BCG3645/46_LF_CHK_R | CAATTGAGTCATCCAGCG |  |

**Supplementary Table S1 Primers used to amplify transposon junctions (5’ to 3’).**

Red sections indicate the P5 or P7-index. The blue section an offset base(s) to ensure that the Illumina sequencer remains accurate while sequencing the tranposon binding section of the primer. The green section binds the transposon. Lower case bases are linked with phosphorothioate bonds. PHO indicates phosphorylation.

Table 2a.

| **BCG Pasteur 1173P2 Position** | **BCG Pasteur 1173P2 Reference genome** | **BCG Danish variant** | **Mutation Type** | **Codon_Subst** | **Amino Acid Subst** | **Locus** | ***In vitro* fold change**  **(log_2_ Danish/ Pasteur)** |
| --- | --- | --- | --- | --- | --- | --- | --- |
| 623418 | C | T | Stop Gained | Cag/Tag | Q323* | galE2 |  |
| 884450 | AG | G | Frame shift | cag/ | Q91 | phoR | 5.00 |
| 1571622 | GC | C | Frame shift | gcc/ | A152 | BCG1434*^1^ |  |
| 1758485 | C | CTCCTCCGGCA | Frame shift | tcg/TCCTCCGGCAtcg | S18SSGI? | BCG1596*^2^ |  |
| 3215013 | AC | C | Frame shift | ata/ | I1780 | ppsC | -2.94 |
| 68923 | A | G | Non-synonymous coding | cTg/cCg | L186P | BCG0067c |  |
| 118865 | T | C | Non-synonymous coding | Tgt/Cgt | C35R | BCG0113 |  |
| 688192 | C | T | Non-synonymous coding | gGc/gAc | G57D | BCG0610c |  |
| 759614 | T | C | Non-synonymous coding | Atg/Gtg | M184V | echA3 |  |
| 1324458 | T | C | Non-synonymous coding | cTc/cCc | L106P | narJ |  |
| 1621517 | T | C | Non-synonymous coding | Tac/Cac | Y251H | whiA | 4.09 |
| 1621518 | A | C | Non-synonymous coding | tAc/tCc | Y251S | BCG1482 |  |
| 1848804 | T | C | Non-synonymous coding | Aac/Gac | N46D | BCG1675c |  |
| 1906675 | G | A | Non-synonymous coding | Gac/Aac | D119N | BCG1714 |  |
| 1937867 | G | T | Non-synonymous coding | gCt/gAt | A2D | PPE22 |  |
| 2619880 | T | C | Non-synonymous coding | cAg/cGg | Q79R | hrcA | 3.89 |
| 2734209 | G | A | Non-synonymous coding | atG/atA | M18I | pepN |  |
| 2860207 | A | G | Non-synonymous coding | Tgc/Cgc | C68R | BCG2594c |  |
| 2976282 | T | C | Non-synonymous coding | gTc/gCc | V46A | sigB |  |
| 2984519 | T | C | Non-synonymous coding | gAt/gGt | D15G | nrdR |  |
| 3315469 | A | G | Non-synonymous coding | aTc/aCc | I50T | ilvH |  |
| 3443321 | G | A | Non-synonymous coding | tCg/tTg | S14L | PPE49a |  |
| 3716536 | A | G | Non-synonymous coding | gTg/gCg | V198A | icd1 | 2.32 |
| 3833488 | G | GGCC | Codon change and codon insertion | gcc/gcGGCc | A433AA | BCG3499c |  |
| 3854969 | G | GGTC | Codon change and codon insertion | ggc/gGTCgc | G261GR | cut3 |  |
| 3907860 | A | G | Non-synonymous coding | Aac/Gac | N599D | PE-PGRS53 |  |
| 19283 | T | C | Silent Synonymous Coding | cgT/cgC | R93 | BCG0018 |  |
| 190828 | C | T | Silent Synonymous Coding | gcC/gcT | A21 | BCG0167 |  |
| 705623 | G | A | Silent Synonymous Coding | caC/caT | H392 | PE-PGRS7 |  |
| 705626 | G | C | Silent Synonymous Coding | gcC/gcG | A391 | PE-PGRS7 |  |
| 2442228 | G | A | Silent Synonymous Coding | ggC/ggT | G23 | BCG2215c |  |
| 2764157 | T | A | Silent Synonymous Coding | acA/acT | T177 | BCG2507c |  |
| 3179819 | T | C | Silent Synonymous Coding | ctA/ctG | L133 | ffh |  |
| 3606542 | C | T | Silent Synonymous Coding | gaC/gaT | D208 | BCG3301 |  |
| 3908180 | C | T | Silent Synonymous Coding | ggC/ggT | G705 | PE-PGRS53 |  |
| 4364138 | T | C | Silent Synonymous Coding | cgT/cgC | R93 | BCG3970 |  |
| 370270 | C | T | Not coding |  |  |  |  |
| 372964 | CA | A | Not coding |  |  |  |  |
| 842686 | AG | G | Not coding |  |  |  |  |
| 1344671 | C | G | Not coding |  |  |  |  |
| 1344672 | G | C | Not coding |  |  |  |  |
| 1383870 | GA | A | Not coding |  |  |  |  |
| 2143356 | T | G | Not coding |  |  |  |  |
| 2143357 | A | G | Not coding |  |  |  |  |
| 3237056 | G | T | Not coding |  |  |  |  |
| 3452980 | G | A | Not coding |  |  |  |  |

Table 2b

| **BCG Pasteur 1173P2 Position** | **BCG Pasteur 1173P2 Reference genome** | **BCG Pasteur variant** | **Mutation Type** | **Codon_Subst** | **Amino Acid Subst** | **Locus** | ***In vitro* fold change**  **(log_2_ Pasteur/Danish)** |
| --- | --- | --- | --- | --- | --- | --- | --- |
| 3833488 | G | GGCC | Codon change and codon insertion | gcc/gcGGCc | A433AA | BCG_3499c |  |
| 3854969 | G | GGTC | Codon change and codon insertion | ggc/ggTCGc | G261GR | cut3 |  |
| 3907860 | A | G | Non-synonymous coding | Aac/Gac | N599D | PE_PGRS53 |  |
| 705623 | G | A | Silent Synonymous Coding | caC/caT | H392 | PE_PGRS7 |  |
| 705626 | G | C | Silent Synonymous Coding | gcC/gcG | A391 | PE_PGRS7 |  |
| 2764157 | T | A | Silent Synonymous Coding | acA/acT | T177 | BCG_2507c |  |
| 1344671 | C | G | Not coding |  |  |  |  |
| 1344672 | G | C | Not coding |  |  |  |  |

**Supplementary Table S2. Genetic differences between BCG Pasteur and BCG Danish and the BCG Pasteur 1173P2 Reference Genome**

Genomic differences between the BCG Pasteur 1173P2 reference genome and the BCG Danish (Table 2a) and the BCG Pasteur (Table 2b) strains used in these experiments. Many of these differences have been noted previously by others [26]. Fold changes are generated by comparing input libraries for BCG Danish and Pasteur, values are only reported for genes with Qvalues < 0.05.

*^1^ Exists as a single gene in *M. bovis* BCG Pasteur. In *Mycobacterium bovis* and BCG Danish, a frameshift due to a single base insertion (*-c) splits the BCG_1434 orthologs into Mb1407 and Mb1408.

*^2^ *M. bovis* and *M. bovis* BCG Danish orthologs share the 10 bp insertion in BCG_1596.

**Supplementary Fig S1a . Accumulation curves for unique TA insertion sites for the input and output BCG Pasteur libraries.**

**Supplementary Data Fig S1b. Accumulation curves for unique TA insertion sites for the input and output BCG Danish libraries.**


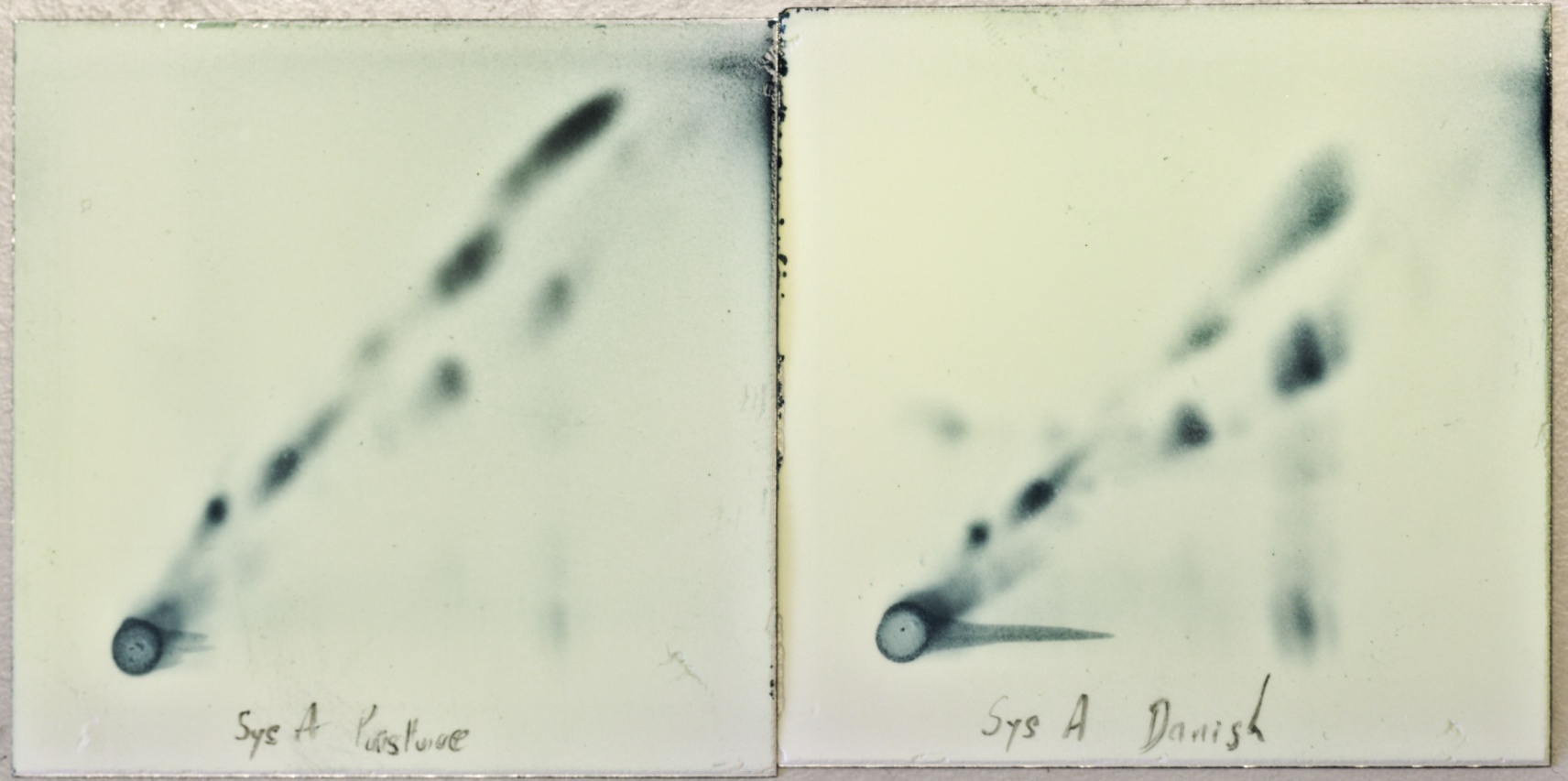


pDIM

Direction II

**Supplementary Fig S2. BCG Danish contains less pDIMs than BCG Pasteur.**

Apolar lipids from BCG Danish and BCG Pasteur were extracted and separated by 2D TLC and visualised by charring after MPA staining.

Direction I

pDIM
